# Australian Cerebral Palsy Child Study: protocol of a prospective population based study of motor and brain development of preschool aged children with cerebral palsy

**DOI:** 10.1186/1471-2377-13-57

**Published:** 2013-06-11

**Authors:** Roslyn N Boyd, Rachel Jordan, Laura Pareezer, Anne Moodie, Christine Finn, Belinda Luther, Evyn Arnfield, Aaron Pym, Alex Craven, Paula Beall, Kelly Weir, Megan Kentish, Meredith Wynter, Robert Ware, Michael Fahey, Barry Rawicki, Lynne McKinlay, Andrea Guzzetta

**Affiliations:** 1 Queensland Cerebral Palsy and Rehabilitation Research Centre, School of Medicine, Faculty of Health Sciences, The University of Queensland, Brisbane, Australia; 2 Department of Rehabilitation, Queensland Cerebral Palsy Health Service, Royal Children’s Hospital, Brisbane, Herston, Australia; 3 Department of Rehabilitation, The Royal Children’s Hospital, Melbourne, Australia; 4 Department of Paediatrics, Monash University, Clayton, VIC, Australia; 5 Queensland Children’s Medical Research Institute, The University of Queensland, Queensland, Australia; 6 School of Population Health, The University of Queensland, Queensland, Australia; 7 Department of Developmental Neuroscience, Stella Maris Scientific Institute, Pisa, Italy; 8 Queensland Cerebral Palsy and Rehabilitation Research Centre, Royal Brisbane and Women’s Hospital, Level 7, Block 6, Herston, QLD, 4029, Australia

**Keywords:** Cerebral palsy, Protocol, Longitudinal cohort, Motor development, Brain structure and function, Communication, Hip displacement, Preschool age, Gross motor function

## Abstract

**Background:**

Cerebral palsy (CP) results from a static brain lesion during pregnancy or early life and remains the most common cause of physical disability in children (1 in 500). While the brain lesion is static, the physical manifestations and medical issues may progress resulting in altered motor patterns. To date, there are no prospective longitudinal studies of CP that follow a birth cohort to track early gross and fine motor development and use Magnetic Resonance Imaging (MRI) to determine the anatomical pattern and likely timing of the brain lesion. Existing studies do not consider treatment costs and outcomes. This study aims to determine the pathway(s) to motor outcome from diagnosis at 18 months corrected age (c.a.) to outcome at 5 years in relation to the nature of the brain lesion (using structural MRI).

**Methods:**

This prospective cohort study aims to recruit a total of 240 children diagnosed with CP born in Victoria (birth years 2004 and 2005) and Queensland (birth years 2006–2009). Children can enter the study at any time between 18 months to 5 years of age and will be assessed at 18, 24, 30, 36, 48 and 60 months c.a. Outcomes include gross motor function (GMFM-66 & GMFM-88), Gross Motor Function Classification System (GMFCS); musculoskeletal development (hip displacement, spasticity, muscle contracture), upper limb function (Manual Ability Classification System), communication difficulties using Communication and Symbolic Behaviour Scales-Developmental Profile (CSBS-DP), participation using the Paediatric Evaluation of Disability Inventory (PEDI), parent reported quality of life and classification of medical and allied health resource use and determination of the aetiology of CP using clinical evaluation combined with MRI. The relationship between the pathways to motor outcome and the nature of the brain lesion will be analysed using multiple methods including non-linear modelling, multilevel mixed-effects models and generalised estimating equations.

**Discussion:**

This protocol describes a large population-based study of early motor development and brain structure in a representative sample of preschool aged children with CP, using direct clinical assessment. The results of this study will be published in peer reviewed journals and presented at relevant international conferences.

**Trial registration:**

Australia and New Zealand Clinical Trials Register (ACTRN1261200169820)

## Background

Cerebral Palsy (CP) is a disorder of movement and posture secondary to an insult to the developing brain [1]. The insult is static and permanent and may be the consequence of different factors, including both genetic and environmental causes. Although the insult is static, the consequent symptoms are variable and may change over time [2]. Children may have a range of associated disabilities, including intellectual disability, hearing and visual deficits, nutritional and feeding problems, respiratory infections and epilepsy [3,4]. Secondary musculoskeletal disorders involving muscle, tendons, bones and joints are common as a result of spasticity, muscle weakness and immobility. CP has substantial lifelong effects on daily function, societal participation and quality of life (QOL) for children and their families.

Cerebral Palsy registers have provided us with some understanding of the aetiologies of CP and specific outcome studies [3]. Few studies have documented broad clinical outcomes for an entire cohort of children with CP prospectively. In addition, none of the existing cohort studies have utilised their large patient groups to better understand the aetiologies of CP, the relationship between abnormalities on brain MRI and outcomes such as motor disability [5] musculoskeletal deformity and related development (communication, oromotor, fine motor skills). A better understanding of the aetiology of CP, the timing of the insult during brain development and the anatomical pattern of injury or malformation is required in order separate CP into different prognostic or treatment groups and to determine the pathway to motor outcome.

Previous studies [5-8] have reported the relative proportions of GMFCS levels (GMFCS I: 27.9-40.7%, GMFCS II: 12.2%-18.6%, GMFCS III: 13.8%-18.6%, GMFCS IV: 11.4%-20.9%, GMFCS V: 15.6%-20.5%), motor types (spastic: 78.2-86.4%, dyskinetic: 1.5%-6.1%, mixed: 6.5%-9.1%, ataxia: 2.5%-2.8%, hypotonia: 2.8%-4.1%), and motor topography (hemiplegia: 15.3%-40.0%, diplegia: 28.0%-46.4%, quadriplegia: 13.6%-50.8%) within various CP cohorts [6,9,10]. A recent systematic review investigating the rates of co-occurring impairments, diseases and functional limitations in CP concluded that for children diagnosed at 5 years of age: 3 in 4 were in pain; 1 in 2 had an intellectual disability; 1 in 3 could not walk; 1 in 3 had hip displacement; 1 in 4 could not talk; 1 in 4 had epilepsy; 1 in 4 had a behaviour disorder; 1 in 4 had bladder control problems; 1 in 5 had a sleep disorder; 1 in 5 dribbled; 1 in 10 were blind; 1 in 15 were tube fed; and 1 in 25 were deaf [4]. Launched in 2007, the Australian Cerebral Palsy Register [3] combines data from several notable state-wide registries (including Queensland, Victoria, Western Australia and New South Wales), and is one of the largest CP registers in the world with over 3,000 children registered in the 1993–2003 birth cohort.

Hip displacement is the second most common musculoskeletal problem in children with CP [11-14]. In the most severely impaired, non-ambulatory children, the incidence may be as high as 80% [11,15]. While children with CP are born with enlocated hips, progression to hip displacement is demonstrated in some children with CP from a very early age [13,14,16]. Hip surveillance programs and appropriately-timed interventions improve outcomes at skeletal maturity [14,15]. Although the final outcome of early intervention at skeletal maturity is not clear [17,18], early risk assessment might enable earlier referral for those children who may benefit from preventative intervention [19]. As clinical assessment of hip range of motion is a poor predictor of risk, several radiological and clinical measures are used to diagnose and monitor hip subluxation [13,16,17,19]. While functional disability, pain [20] and impaired ambulatory weight-bearing [12,16,18,19] are associated with risk of hip displacement and need for surgical intervention, the evidence regarding radiological characteristics is less clear [21,22]. There is a need for early prospective evaluation of radiological development in a population of very young children with CP across the spectrum of function severity in order to aid prediction of hip development.

There have been several large studies that have evaluated prospective motor development in children with CP. The Ontario Motor Study (OMGS) collated over 2,632 GMFM assessments on 657 children with an average of four observations per child [9]. The principal outcome of the study was the development of two internationally accepted valid and reliable tools for measuring motor function (the Gross Motor Function Measure, GMFM) [9,23] and for classifying functional status into five groups (Gross Motor Function Classification System, GMFCS) [24,25]. From these data, Growth Motor curves for children with CP were developed [9]. These curves are valid and reliable for children aged two years and over and allow for tracking and predicting motor outcomes for children by GMFCS classification [25]. Two potential limitations of the Ontario Motor Study were that it included only minimal data on children less than 3 years of age and it was a not an entire population based sample [9].

In the European Cerebral Palsy study [6], with a representative cohort of children with CP from eight European countries, children are classified according to brain injury diagnosed using MRI. This group used a classification system based on the presumed timing and nature of the insult that resulted in CP and included both genetic and non-genetic aetiologies such as genetic cortical malformations (e.g. lissencephaly) and hypoxic ischaemic injury [6,10]. Again this cohort is representative rather than entire population based and these investigators from Surveillance of Cerebral Palsy in Europe (SCPE) have guided our classifications of motor type and of the brain injury on MRI [26-28].

**Figure 1:**
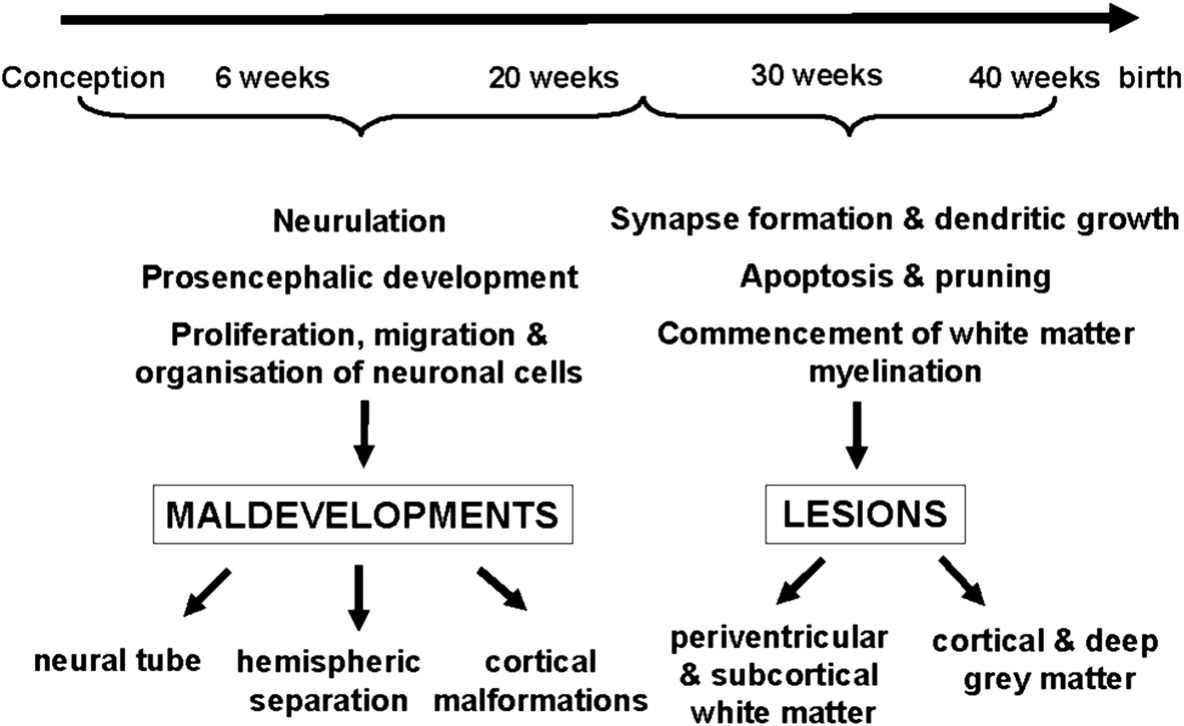
**Major events in human brain development.** Pathogenic events (both genetic and non-genetic) affect the developing brain to cause malformations or lesions, the patterns of which will depend on the stage of brain development during which the event occurs.

Pathogenic events impacting on the brain cause different patterns of structural abnormality in CP [29]. These pathogenic events may be environmental or genetic. Their consequences will depend not only on the nature of the event, but also the timing of the event during the different stages of brain development (Figure 1). The 1st and 2nd trimesters are the most critical times for cortical development and are characterized by the sequential yet overlapping steps of proliferation, migration and organization of neuronal cells and their connections. Brain pathology secondary to events during these stages of brain development is usually characterised by significant malformations. During the 3rd trimester, growth and differentiation events are predominant and persist into postnatal life. Disturbances of brain development during this period cause lesions, often of a different pattern to those resulting from earlier insults or developmental disorders. During the early 3rd trimester, the periventricular white matter is especially affected; whereas towards the end of the 3rd trimester grey matter, either cortical or deep grey matter, appears to be more vulnerable. Understanding the aetiologies of CP in the living patient has advanced significantly since the increased use of MRI in the evaluation of children with congenital or early-onset neurological deficits. Using MRI, a number of studies have shown that the most common causes of CP are structural brain lesions [27,30-33], especially prematurity-related injuries, and malformations of brain development [34-36]. Guidelines by the American Academy of Neurology strongly recommend that all children with a suspected diagnosis of CP undergo neuroimaging, with MRI preferable to CT [37]. Determination of brain structural abnormality will provide a final diagnosis that is more than a label of ‘cerebral palsy’[38].

It is necessary to attempt to determine the underlying aetiology/pathogenesis to confirm the suspicion of a static lesion, exclude a treatable disorder and diagnose a malformation, which may have significant genetic counselling implications for the family. In addition, these patterns of brain maldevelopments or lesions offer excellent models to study the normal mechanisms of organisation and reorganisation in the developing brain [30,31,39]. Despite these advances, limited studies exist correlating the specific MR imaging appearance and outcome measures such as motor function [27]. Such data may prove invaluable in providing accurate prognostic counselling at the time of diagnosis, as well as potentially guiding the most appropriate treatments tailored to each individual’s pattern of CP and type of lesion on imaging.

A recent systematic review investigated the relationship between brain structure on MRI and motor outcomes in children with CP [40]. A total of 37 studies comprising over 2300 subjects met inclusion criteria, and these studies were analysed in terms of population characteristics, MRI data, motor outcome data, and where possible, the relationship between MRI data and motor outcomes. The importance of MRI lesion description has been previously outlined, due to the presumed relationships between lesion topography and motor type, and between lesion extent and functional severity [27]. Indeed, Yokochi et al. [29] and Holmstrom et al. [41] reported that in subjects with motor subtypes of athetosis or hemiplegia respectively, motor disabilities were more severe when lesions involved both grey and white matter on MRI as opposed to grey or white matter involvement alone. Similarly, Holmefur et al. [42] reported that in subjects with spastic hemiplegia, those with more severe white matter reduction on MRI had a significantly lower development in hand function. A focus of current research is the prevention of CP, which requires clinical outcomes to be correlated with the presumed timing and aetiology of lesions in the developing brain [43]. Pathological insults during brain development cause abnormalities or lesions which may be detected by brain MRI, and the observable patterns of these lesions depend on the stage of brain development [39]. Using this principle, a qualitative classification system has emerged whereby lesions can be identified as brain maldevelopments, periventricular white matter lesions, grey matter lesions, other miscellaneous lesions, or normal MRI [27]. All studies included in the review reported enough MRI data for subjects to be classified into these broad lesion groups, and differences in motor subtypes and functional disabilities were identified between groups [40]. Despite this, it was found that many studies did not utilise valid and reliable classifications and measures of motor abilities (e.g. GMFCS, GMFM, and MACS), and heterogeneous measures were employed which generally precluded pooled analysis. All included studies also used a qualitative system of lesion description or classification [27], and as such the specific anatomical location and severity of brain pathology was often overlooked. Ultimately, the authors concluded that the relationship between MRI findings and motor outcomes needs to be further investigated in a cohort of children with CP using a valid, quantitative measure of MRI classification which includes detailed information about the location and extent of brain lesions, as well as valid and reliable motor measures [40,44].

**Figure 2:**
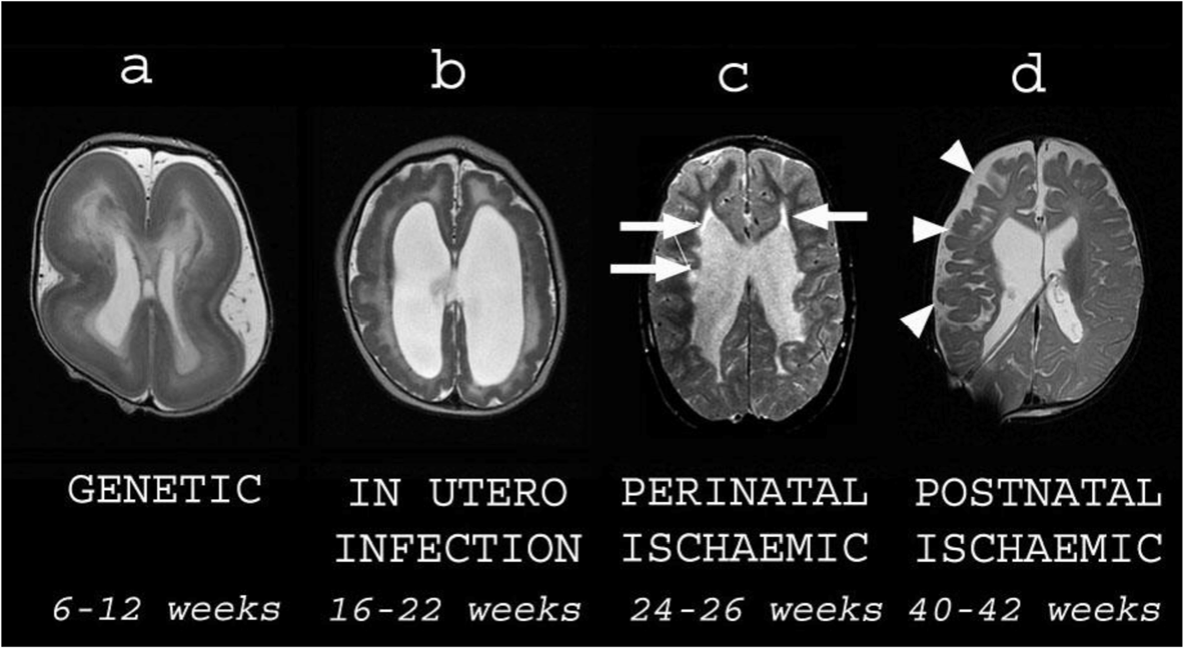
**Examples of different types of structural brain abnormalities in cerebral palsy All images are axial T2-weighted MRI scans.** Each image is subtitled by its presumed aetiology and timing during gestation. **a** is a child with lissencephaly showing cortical thickening and agyria. **b** is a child with congenital cytomegalovirus infection showing an overfolded cortex (polymicrogyria), thin white matter and dilated lateral ventricles. **c** is an ex premature child showing cystic white matter injury (arrows) consistent with periventricular leukomalacia. **d** is a child who suffered a haemorrhagic stroke in the newborn period. There is cortical and white matter loss in the right frontal and parietal lobes (arrowheads) consistent with previous ischaemia.

The limitation of many cohort studies of children with CP in Canada [9], the USA, and across Europe [10] is the difficulty obtaining a representative sample and an entire cohort. The opportunity for undertaking entire prospective cohort based studies is possible in Australia. There is limited data on motor trajectories of an entire cohort of children with CP from diagnosis at 18 months to 36 months of age and these motor trajectories have not been correlated with MRI brain injury classification. For the present study the age of 18–24 months for entry has been chosen as diagnosis is usually confirmed by this time. Children will be followed up till 5 years of age at school entry when motor outcome has been well classified [3]. The preferred age for structural MR imaging is from 24 months because by this age myelination of the brain should be complete, thus allowing optimum differentiation between grey and white matter on MR imaging, important for the detection and correct classification of brain injuries and malformations (Figure 2).

**Figure 3:**
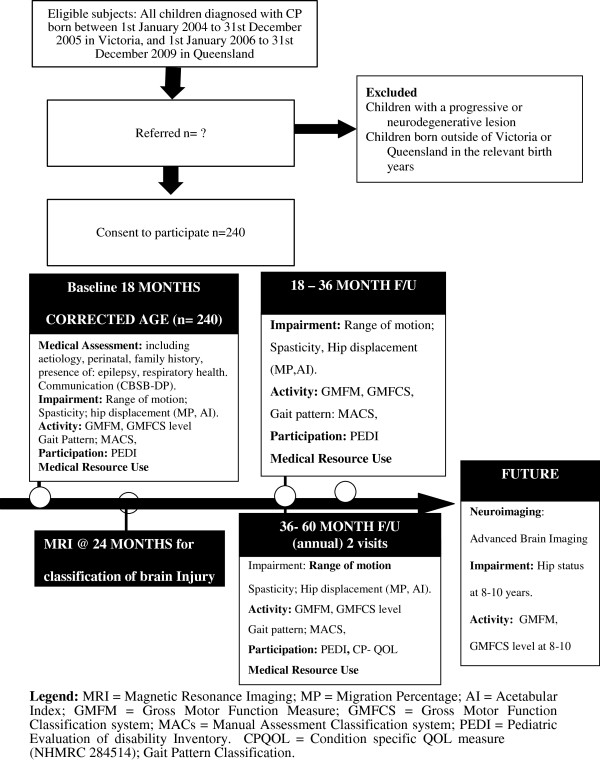
Consort flowchart of study program.

In the Australian CP child study (NHMRC 465128) entire birth years of Victorian and Queensland born children with CP are prospectively entered and will be followed intensively to determine the relationship between the rate and limit of motor development (gross and fine motor function) as related to the nature of the brain lesion. Secondarily the influence of musculoskeletal deformity (hip displacement, spasticity and muscle contracture) and location and extent of brain injury will be related to the rate and pattern of motor disability. The parent report of their child’s ability to participate in society and perceived quality of life will be compared across motor severity. Finally the level of motor functioning will be correlated with direct medical and allied health costs and outcomes including school readiness (see study flow chart, Figure 3). School readiness is a framework for assessing profiles of strengths and vulnerabilities of the preschool aged child [45]. It considers a child’s readiness to learn within five major skill areas: health and physical development, emotional well-being and social competence, approaches to learning, communication skills, and cognitive skills and general knowledge [45].

### Aims and hypotheses

This study aims to determine the pathway(s) to motor outcome (gross and fine motor) from diagnosis at 18 months to outcome at 5 years in relation to the nature of the brain lesion (using structural MRI). These aims will be explored through the following hypotheses:

1 The rate of motor development (gross motor function) from 18 months will be related to the limit of attainment at 5 years (Gross Motor Function Classification, GMFCS level).

2a The pattern of motor disability (motor type and distribution) will correlate with the location, presumed timing and nature of the brain lesion(s).

2b The severity of motor disability in CP (age of onset or signs) will correlate with the location, extent and nature of the brain lesion (on structural MRI).

3 The rate and limit of motor development will be influenced by the severity of musculoskeletal deformity (i.e. slower motor development will correlate with marked hip displacement, increased spasticity and reduced range of motion in the lower limb).

4 Children with lower levels of function will have higher direct medical and allied health costs.

### Study significance

#### This unique project will

1. Allow clinicians to better predict the functional outcomes of children with CP from an earlier age based on their rate and limit of gross motor abilities and nature and severity of their brain lesion.

2. Determine the nature and timing of physical deformities including hip displacement to guide the timing and intensity of interventions.

3. Provide comprehensive data on the relationship between the nature of the brain lesion, rate of musculoskeletal deformity and impact on the child’s ability to participate in the community.

4. Information on resource use for future planning of medical and therapy services.

## Methods

All children diagnosed with CP, born in the years 1^st^ January, 2004 to 31st December, 2005 in Victoria, Australia and 1^st^ January 2006 till 31^st^ December, 2009 born in Queensland, Australia will be entered (n = 240). We define Cerebral Palsy as a permanent (but not unchanging) disorder of movement and posture that results from an insult to the developing central nervous system. The characteristic signs are spasticity, movement disorders, muscle weakness, ataxia and rigidity [43].

### Exclusion criteria

1. Children with a progressive or neurodegenerative lesion.

2. Children born outside of Victoria or Queensland in the relevant birth years.

### Ethics approvals

Ethics committee approvals have been gained through The Royal Children’s Hospital Melbourne Ethics Committee, (HREC/25010 F), Southern Health Human Research Ethics Committee C (05077C), University of Queensland Medical Research Ethics Committee (2007001784), the Children’s Health Services District Ethics Committee (HREC/07/QRCH/107), the Mater Health Services Human Research Ethics Committee (1186C), the Queensland Cerebral Palsy Register at the Cerebral Palsy League of Queensland (CPLQ 2008/ 09–1010), Gold Coast Health Service District Human Research Ethics Committee (HREC/08/QGC/45), Central Queensland Health Services District Human Research Ethics Committee (HREC/08/QCQ/19), Cairns and Hinterland Health Service District Human research Ethics Committee (HREC/08/QCHHS/521) and the Townsville Health Service District Human Research Ethics Committee (HREC/08/QTHS/33). There are no known health or safety risks associated with participation in any aspect of the described study. All families will give written informed consent to participate, and they are able to withdraw their child from the study at any time without explanation, without any penalty from staff at the Royal Children’s Hospital or University of Queensland, or any effect on their child’s care. Data collected in this study will be stored in a coded re-identifiable form (by ID number). Each child has multiple assessment appointments across the duration of the study, which necessitates data to be re-identifiable.

### Ascertainment of the cohort

Prospective entry of birth years born in Victoria (born in 2004 and 2005) and Queensland (born in 2006, 2007, 2008, 2009) entered at 18 months will be followed until school age (5 years) (n = 240-360). Study recruitment commenced in July 2005 (at 18 months c.a.) for children born in January 2004 and continues in Queensland according the above birth years.

State wide recruitment has been established in collaboration with the relevant Cerebral Palsy Registers with data collection at tertiary referral hospitals. Community awareness has been generated through campaigns aimed at paediatricians (Division of Paediatrics & Child Health), general practitioners, allied health professionals, maternal and child health nurses, and neonatal follow-up clinics. These groups have been encouraged to refer children with motor delay (not sitting at 10 months, not standing at 12 months not walking at 24 months) for confirmation of a diagnosis of cerebral palsy. Families of children identified through the relevant CP Register have been approached after permission to contact the family has been given by their treating clinician or direct referral to the study by families whom have provided consent to be entered onto the Queensland CP Register (QCPR). Specialist clinics have been established at the tertiary referral centres where suitability for the study can be confirmed. In cases where the diagnosis of CP is unclear, or where there is a suggestion of a progressive or degenerative course, further investigations (such as metabolic screening) will be requested before a diagnosis of CP is confirmed. Parents have then been invited to participate in the study and give informed consent. High ascertainment is expected for children with moderate to marked motor delay (GMFCS III to IV) and this has been the case for children born preterm and children referred to surveillance clinics at tertiary referral centres. Children born at term with mild motor delay (GMFCS level I, II) and predominant lower limb involvement (diplegia) are typically identified through the CP orthopaedic services and spasticity management clinics. Children with hemiplegia (GMFCS level I and II) are detected early through the surveillance clinics and occupational therapy services. Children who are detected after 18 months of age will be entered into the study at the time of diagnosis, will be offered brain MRI at entry and be followed up with serial motor assessments and other outcomes until outcome at 5 years.

### Measurements and procedures

Following confirmation of a diagnosis of CP, eligible children are entered from 18 months corrected age. They will be assessed for diagnostic criteria, co-morbidities and for differential diagnosis by neurological assessment (by a Paediatrician, Child Neurologist or Paediatric Rehabilitation Specialist). Experienced Physiotherapy researchers will perform all GMFM assessments adjacent to either clinic visit and perform collection of range of motion, clinical measures of spasticity, then rate GMFCS, gait pattern, MACs and measures of pelvic radiographs according to standardized protocols.

### Primary measures

The aim of the present study is to gather information regarding the longitudinal measurement of Gross Motor Function (GMFM-66) from 18 months to 5 years [46] and determine the aetiology of CP using clinical evaluation combined with MRI (location, nature and structure of the brain lesion) [27]. The lesion will be classified by 3 main criteria:

A. the *anatomical features* of the lesion:

i. localisation by tissue (e.g. cortical, white matter, deep grey matter etc.)

ii. localisation by region (e.g. lobes involved, laterality etc.)

iii. extent of lesion (e.g. generalised, hemispheric, lobar etc.)

B. the presumed *aetiology* of the lesion: (i) genetic; (ii) ischemic; (iii) infective and (iv) other.

C. the presumed *timing* of the insult that caused the lesion:

i. Prenatal by trimester or by stage of brain development;

ii. Perinatal;

iii. Postnatal.

All MRIs will be classified by a neurologist together with a neuroradiologist using a standardised method of image evaluation and classification. Following these evaluations, consensus will be reached regarding the above three criteria. We estimate that 70–80 percent of children currently receiving a diagnosis of CP will have had brain MRI as part of their clinical work-up. The American Academy of Neurology has concluded that a brain MRI should be part of the diagnosis of CP in a previous practice parameter [37]. For Victorian patients, the majority will have had their imaging performed and reported through the Royal Children’s Hospital, Melbourne or Monash Children’s Hospital Medical Imaging Department on a GE Signa Echo Speed 1.5T MR scanner. For Queensland patients, the majority will have had their imaging performed and reported through the Royal Children’s Hospital, Brisbane Medical Imaging department on a GE Signa Echo Speed 1.5T MR scanner. The current minimum imaging protocol for patients with suspected CP consists of axial fast spin echo and coronal fast spin echo sequences and 3D inversion prepared fast spoiled GRASS sequence. 3D acquisitions are reformatted in axial, coronal and sagittal planes, with additional oblique and curved reformatting. Age specific protocols are used to maximize the ability to detect cortical and white matter abnormalities at different stages of myelination. All existing neuroimaging will be re-reviewed by a neurologist familiar with the features of lesions that result in CP, most commonly either white matter injury or congenital malformations. A protocol will be used to describe the features of each patient’s abnormality. The patient’s imaging will then be classified using a system, which takes into account anatomical features, aetiology and presumed timing of the “insult” causing the abnormalities. If no MR imaging has been performed, or if previous imaging was only CT scans or poor quality MRI scans, then an attempt will be made to perform high quality MR imaging. Such imaging will usually be necessary for clinical reasons to be able to make an accurate diagnosis and exclude causes of CP that may have genetic implications for other family members. This approach is consistent with recent guidelines suggesting that all patients with the label of CP have high quality MR imaging on at least one occasion [37]. For children scanned prospectively, this will be performed at the either Paediatric Magnetic Resonance Imaging Centres. All MRI scans will be performed clinically under anaesthesia after informed consent.

Brain lesion severity will be assessed using a structured scoring proforma [44] based on the CH2 template [47], a highly detailed single-subject T1 template in MNI space, which is the international standard for brain mapping (International Consortium of Brain Mapping - ICBM). Lesions will be transcribed onto the proforma and the following measures obtained: number of (i) anatomical lobes involved, (ii) number of slices on the template that were affected and (iii) size and distribution of the lesion measured by a global lesion score and lesion subscores. The number of lobes and slices affected will be the average of summed right and left hemispheres. To calculate total lesion score, each frontal, parietal, temporal and occipital lobe will be first considered in three sections: periventricular, middle and subcortical matter. Each section will be scored as 0.5 if less than 50% of area was involved; or 1, for greater than 50% involvement, with a maximum lobar score of 3. Lobar scores for each hemisphere will be summed, with a maximum hemispherical score of 12 possible. The total lesion score will be the sum of right and left hemispherical scores (maximum score 24). A 1-point score (involved/not involved) will also be attributed to 16 anatomical structures including the corpus callosum, the cerebellum and the main subcortical structures. The final maximum score of the scale will therefore be 40 (24 + 16).

#### Gross motor function

At each assessment gross motor function is evaluated using the GMFM-66 & GMFM-88 [46]. The GMFM-88 assesses childrens’ motor abilities in lying to rolling, sitting, crawling to kneeling, standing, walking, running and jumping. The GMFM-66 is comprised of a subset of the 88 items identified (through Rasch analysis) as contributing to the measure of gross motor function in children with cerebral palsy. The GMFM-66 will be used to provide an overall measure of gross motor function and the GMFM-88 domain scores to explore specific motor skills [46]. Measures of GMFM will be rated by experienced research physiotherapists.

### Secondary measures

#### Gross motor function classification system (GMFCS)

The Gross Motor Function Classification System (GMFCS) is a five level classification system of children’s functional gross motor severity. It is based on self-initiated movements, anti-gravity postures and motor skills expected in a typical five year old [25,26]. Children who are independently ambulant are classified as GMFCS I or II, those requiring an assistive mobility device to walk classified as GMFCS III and those in wheeled mobility as GMFCS IV and V. Two physiotherapists, trained in the use of the GMFCS, independently observe and classify children in one of five functional categories [25]. The GMFCS has internationally established validity, reliability and stability for the classification and prediction of motor function of children with CP aged 2–12 years [24,25]. It has a high inter-rater reliability (generalisability coefficient = 0.93) [25]. Classifications of gross motor abilities change with age, therefore separate descriptions are used for different age bands. In the current study, the <2 years and 2–4 year descriptions are used. Lower inter-rater reliability is documented for the <2 years age band (κ = 0.55), as younger children’s gross motor abilities are more variable, and less developmental information is available on which to base the classification [48]. The intra-rater (test retest) reliability from <2-12 years appeared to be acceptable (generalisability coefficient = 0.68). The GMFCS has been correlated with a number of motor scales, as well as CP distribution and type of motor impairment [49].

#### Motor type & distribution

Motor type of CP will be classified as spastic, dystonic, ataxic, hypotonic, choreoathetosis, mixed CP or unclassifiable according to SCPE guidelines [28,50]. Distribution will classified by number of limbs impaired (hemiplegia, diplegia, triplegia, quadriplegia) by at least two independent raters [51].

#### Motor performance

Functional performance will be scored on the Functional Mobility Scale (FMS). This is a valid and reliable measure of a child’s usual walking ability at three distances (5 m, 50 m and 500 m), representing their home, school and wider community [52].

#### Gait pattern classification

Gait patterns will be classified according to the Rodda & Graham’s Classification for spastic diplegia [53,54], which has demonstrated validity and reliability [53]. From least to most severe these were: (i) True Equinus, (ii) Jump Knee, (iii) Apparent Equinus and (iv) Crouch Gait. For children with unilateral CP, gait patterns will be classified according to Winters & Gage [55]. This classification considers the sagittal plane joint movements. Group I: foot drop during swing phase (Apparent Equinus). Group II: persistent ankle dorsiflexion (True Equinus). Group III: maintained plantar flexion through gait cycle plus limited knee flexion-extension. Group IV: similar to III, plus reduced hip flexion-extension [53,56]. Winter’s classification [55] has good inter-rater reliability using written reports (weighted kappa, wκ = 0.76) and videos (wκ = 0.63) [57,58].

#### Upper limb function

Upper limb function is classified using the Manual Ability Classification system (MACs) [59]. The MACs is an international system to classify hand function based on the child’s typical performance when handling objects in daily activities. This classification system was developed for children aged from 4–18 years, but has been shown to have good reliability for use in children as young as two years [59].

#### Radiological measures of hip displacement

Hip surveillance, including anterior-posterior (AP) pelvis x-ray, is recommended for all Australian children with CP to facilitate early detection and treatment of severe or progressive hip displacement [14,60,61]. The migration percentage (MP) is widely accepted as the gold standard measure in hip surveillance [12,62], measuring femoral head subluxation. Other measures include the acetabular index (AI), assessing acetabular dysplasia [63], and the femoral neck-shaft angle (NSA) [64,65]. As the pelvis and its radiographic appearance changes between birth and skeletal maturity [66], early surveillance may be impacted by bony growth and ossification, particularly if measurements are based on landmarks that are difficult to identify or absent in the immature skeleton. The reliability of migration percentage has been investigated in relatively small studies to date [67,68], and reliability data in very young children is infrequent. Hilgenreiner’s Epiphyseal Angle (HEA) [69] is a radiographic measure describing the proximal femoral epiphysis and has been previously applied to assessment of coxa valga [70,71], but may offer prognostic information for hips at risk in cerebral palsy. It is the acute angle between a line drawn parallel to and through the proximal femoral epiphysis and Hilgenreiner’s line [69].

#### Musculoskeletal development

A comprehensive musculoskeletal examination will be performed by paediatric physiotherapists recording data relating to joint range of movement, muscle length, leg length difference, bony anomalies, motor type and muscle contracture.

#### Clinical history and examination

At study entry including a comprehensive clinical history and examination at study entry is performed by a paediatrician, child neurologist or rehabilitation physician. The following information is collected:

a. Presence or absence of vision impairment, hearing difficulties; epilepsy;

b. Feeding issues including presence or absence of gastrostomy tube and failure to thrive;

c. Respiratory difficulties including episodes of pneumonia and aspiration;

d. Speech and language development.

#### Participation

Children’s participation will be assessed (i) via parent-report on the domains of self-care, mobility and social functioning using the scaled scores of the Paediatric Evaluation of Disability Inventory (PEDI) which has good validity and reliability [72-74] and (ii) parent perception of health related quality of life using a condition specific tool the CPQOL-child by parent report [75,76] at 5 years.

#### Medical and allied health resource use

In order to determine the relationship between motor prognosis and medical and allied health resource use, the direct costs of treatment will be monitored and compared to outcomes with adjustment for confounders such as disease severity.

#### Communication

Communication difficulties will be examined by parent self-report on the Communication and Symbolic Behaviour Scales–Developmental Profile (CSBS-DP) Infant-Toddler Checklist [77,78] (24 parent rated items) and the Communication Function Classification System (CFCS) [79]. The CSBS-DP screening tool is a parent questionnaire comprised of three composite subtests: social, speech and symbolic, and a total score. The social composite, composed of 13 questions, investigates the child’s ability to functionally communicate, use eye gaze and gesture. The speech composite, comprising five questions, examines the sounds and words the child uses and their ability to combine words. The symbolic composite, comprising of six questions, explores the child’s understanding of language and their ability to appropriately use objects such as a cup, spoon, toy telephone, stacking blocks, and participation in pretend play. Raw scores for each composite were converted into standardized scores (SS) where the M = 10 (standard deviation, *SD* ± 3). The total score for the CSBS-DP was calculated by adding the raw composite scores, then converting to SS with M =100 (*SD* ± 15) [77]. The CSBS-DP manual recommends all children with SS ≤ six on composites, or ≤ 81 on the total score, be referred for further speech and language evaluation. The CSBS-DP Infant-Toddler Checklist has been shown to have high test-retest reliability (*r* range = 0.79 to 0.88) [77], a strong predictive relationship with expressive and receptive language (*R* = 0.55 and 0.71 respectively) and high sensitivity and specificity (76% and 82% respectively) at two years of age [77,78]. The Communication Function Classification System (CFCS) will be used to classify everyday communication performance of individuals with cerebral palsy into five classification levels [79]. All methods of communication performance are used in assigning the level of function, including both informal (gesture, behaviour), and formal (speech and symbolic communication systems). The classification has good inter-rater reliability, conducted on 69 children aged 2-18 years (0.66 overall, and 0.77 for children older than 4 years), and excellent test-retest reliability (0.82) [79].

*Neurological Examination:* Existing data regarding the child’s neurological examination will be reviewed. Children will receive a comprehensive neurological examination by a rehabilitation specialist, developmental paediatrician or paediatric neurologist. It will be undertaken again if this has not been performed or documented comprehensively by such specialists within the previous six months.

#### Epilepsy

Epilepsy is common in CP, occurring in around 50% of children [80-82]. The presence of poorly controlled epilepsy or excessive anticonvulsant medications may confound an accurate assessment of each child’s clinical state. For this reason we will obtain data on each child’s pattern of epilepsy including age of onset, seizure type, frequency and medications.

### Data analysis plan

A comprehensive database has been established for all data collection, including clinical measures, MRI scoring and questionnaires so that it is entered prospectively at the time of each assessment. Summary reports are automatically generated from the database to report back to families and treating clinicians after each visit. Our biostatistician will supervise the statistical methods proposed in this study, including analysis of binary outcomes in longitudinal studies using weighted estimating equations (e.g. presence of co morbidities); multilevel mixed-effects models of longitudinal binary outcomes (e.g. GMFCS levels), and generalised estimating equations for ordinal data.

For hypothesis I: Raw GMFM total score will be converted to GMFM-66, Rasch analysed scores. The GMFM-66 data will then be plotted by age in months for the entire cohort then according to GMFCS group. Parameters of a non-linear model of motor development will be estimated using non-linear fixed effects modelling for children according to their GMFCS level. The model uses two parameters, the estimated rate and limit of motor development. Other complex, longitudinal analysis methods such as multilevel mixed-effects models and generalised estimating equations [83] will also be employed to look at the temporal relationships between motor trajectories and classifications of brain structure on MRI (Hypothesis 1, 2), and musculoskeletal deformities (Hypothesis 3). For Hypothesis 4 groups of children (by GMFCS level) will be compared economically by incremental cost effectiveness and cost utility ratios.

#### Sample size calculations

For Hypothesis 1 six measurements are planned for each participant between 18 months and 5 years of age. A sample size of 40–50 per group (GMFCS I-V will give a total of 240 patients) for a two-group comparison of slopes in a linear model of motor development will have 80% power [9] of detecting if there is a difference between the GMFM curves based on initial GMFCS groups. This range allows for a range of possible effect sizes (based on results of Rosenbaum et al. [9]), and a range of between- and within-person variability in GMFM measurements over time (allowing for a linear pattern of motor development based on data from our own study of 90 children over 3 years (NHMRC 980753). The initial GMFCS classification is the primary predictor variable and GMFM-66 score at five subsequent time points will measure the pathway to motor outcomes. In the event that children are diagnosed after 18 months corrected age they will be entered at the age of diagnosis and will drop in to the study at entry. Previous ascertainment rates suggest that children will be identified by 2–3 years which would allow a minimum of 3–5 data points for analysis, appropriate for linear modelling.

For Hypothesis 2 for comparisons among MRI classification levels (anticipating 43% PVL brain loss, 16% BG damage, 16% cortical/subcortical, 12% malformation/miscellaneous, and 10% normal from [5], or comparisons among GMFCS levels (anticipating 36% level I, 16% II, 14% III, 16% IV, 18% V: [6]) we need a total cohort of approximately 250 children. For the non-linear model of motor development, sample size calculation is complex however 80 subjects per group with 4 GMFM measurements was sufficient to estimate the asymptotic limit parameter with precision ± 3 GMFM-points (width of 95% confidence interval) in a similar population [9]. A study of approximately 40 per group with 6 measurements will have slightly lower precision for this parameter but should be sufficient for identifying differences between GMFCS groups as the differences are large (>10 GMFM-points) [9].

## Discussion

This study protocol describes the rationale, aims, hypotheses and methods for a large prospective longitudinal population-based study of early motor development and brain structure in a representative sample of preschool aged children with Cerebral Palsy, using direct clinical assessment. The results of this study will be published in peer reviewed journals and presented at relevant international conferences.

## Competing interests

The authors declare that they have no competing interests.

## Authors' contributions

RB is the chief investigator and together with MF and BR conceptualized, designed and established this research study. RB, RJ, AM, CF, BL, PB and MK also contributed to study design and were responsible for the selection of particular assessments. RB, MF, LM and AG were responsible for the brain MRI analysis content. RB, LP were responsible for ethics applications and reporting. RB, RJ, LP, LM, MK, MW will be responsible for recruitment and data collection in Queensland and RB, AM, MF, BR for recruitment and data collection in Victoria. RB drafted the manuscript with input from all the co-authors. All authors have agreed the final version of the manuscript and were involved in the decision to submit the manuscript. There is no financial support for the authors regarding this manuscript. The external funding agencies (NHMRC, Telstra Foundation) have provided funds for the conduct of the study but will not be involved manuscript preparation, decisions to publish or the interpretation of results arising from the study. All authors read and approved the final manuscript.

## Pre-publication history

The pre-publication history for this paper can be accessed here:

http://www.biomedcentral.com/1471-2377/13/57/prepub
